# Inflammatory Markers Significantly Increased in Patients Treated with Obinotuzumab for Lymphoproliferative Diseases

**DOI:** 10.3390/jcm13237146

**Published:** 2024-11-26

**Authors:** Krzysztof Gawronski, Nadia Hussein, Piotr Rzepecki

**Affiliations:** Department of Hematology, Military Institute of Medicine—National Research Institute, Szaserow Street 128, 04-141 Warsaw, Poland; nhussein@wim.mil.pl (N.H.); przepecki@wim.mil.pl (P.R.)

**Keywords:** C-reactive protein CRP, procalcitonin, anti-CD20+ antibodies

## Abstract

**Background**: The purpose of this study was to analyze the behaviors of inflammatory markers, such as procalcitonin and C-reactive protein (CRP), during treatment with obinotuzumab (an anti-CD20 antibody). **Methods**: Our non-randomized observational study prospectively evaluated a cohort of 22 adult patients with lymphoproliferative neoplasms, chronic lymphocytic leukemia (CLL), and follicular lymphoma (FL) with indications for obinotuzumab therapy. **Results**: All patients had their blood drawn to determine blood counts, CRP, and procalcitonin, as well as body temperature measurements and blood cultures performed for bacterial infections on day 0 before administration of the anti-CD20 antibody. Subsequently, on days 1 to 7 after administration, blood was drawn daily at a fixed time of 8:00 a.m. for blood counts and CRP and PCT values, and blood cultures were performed. In addition, on days 1 to 7, body temperature was measured at fixed times (i.e., 8:00 a.m. and 8:00 p.m.). In all of these patients, significant increases in inflammatory markers, such as CRP and procalcitonin, were observed shortly after drug infusion. There was a statistically significant change in the serum PCT concentration (*p* < 0.0001), which significantly increased on days 1 to 4 compared to the initial measurement 0. **Conclusions**: The increases in inflammatory markers shortly after obinotuzumab (anti-CD20 antibody) administration can be significantly high but are most often not related to the onset of infection and do not lead to any ill consequences in the treatment of lymphoproliferative disease.

## 1. Introduction

According to the World Health Organization (WHO), lymphoproliferative diseases and lymphoproliferative disorders (LPDs) are a group of diseases characterized by the uncontrolled production of lymphocytes [1,2,3], often occurring in immunocompromised individuals [4,5]. Lymphoproliferative diseases include chronic lymphocytic leukemia/small lymphocytic lymphoma and follicular lymphoma. Chronic lymphocytic leukemia/small lymphocytic lymphoma (CLL/SLL) belongs to the class of mature B-cell neoplasms. The threshold of less than 5 × 10^9^/L is arbitrary but identifies a group with a very low likelihood of requiring treatment compared to individuals with B-cell counts between 5 and 10 × 10^9^/L [5]. Follicular lymphoma is a neoplasm arising from the B-cells of lymph nodules, and follicular lymphoma is diagnosed most often in people over the age of 60 [5].

Patients with lymphoproliferative diseases treated with chemotherapy and anti-CD20 monoclonal antibodies tend to be in a state of severe immunosuppression, which increases their risk of infection. In this context, clinical and laboratory signs of sepsis are of limited diagnostic value, and the white blood cell (WBC) count is low. Patients with lymphoproliferative diseases have an increased risk of infectious complications related to the fact that various types of immune deficits can enable lymphoproliferative disease development, inducing the production of the monoclonal lymphocyte in pathogenesis, thus leading to a functionally defective immune system [6]. In addition, the use of treatments, such as chemotherapy or immunotherapy, that affect lymphocyte depletion can further exacerbate immunosuppression [7]. All of this can result in an increased propensity to develop severe forms of infections in patients, including sepsis and its complications [8].

Under such therapies, the patient’s white blood cell (WBC) counts may be low, making them of little diagnostic value. Fever, as an important clinical sign, can occur for several reasons or may be completely absent. Rapid detection of neutropenic fever and incipient sepsis in hematologic patients is very important for therapeutic success. In order to detect patients at risk of clinically significant infection, it is important to evaluate biochemical indicators of inflammation, such as C-reactive protein (CRP) and procalcitonin (PCT), which can be reliable indicators for diagnosing infection, but only in the general population. PCT appears to be better for the early detection of inflammation and can distinguish systemic inflammatory response syndrome (SIRS) from sepsis [9]. PCT levels are elevated even in immunosuppressed patients with sepsis, helping to identify those who require antibiotic treatment [6,7,8].

Obinotuzumab, an anti-CD20 antibody, is often used as a part of immunochemotherapy to treat chronic lymphocytic leukemia and other lymphoproliferative syndromes [5]. In these patients, the absence of infection prior to therapy is crucial. The administration of obinotuzumab can be associated with a systemic reaction, including fever—which is very characteristic of sepsis [10,11]—and even symptoms of septicemia (SS), which is a rare form of systemic inflammatory reaction with complex etiology [12]. The depletion of leukocytes—or, more precisely, lymphocytes—induced by obinotuzumab renders one of the key diagnostic criteria for SIRS/sepsis useless, namely, an increase in leukocytosis [13]. Therefore, biochemical markers of inflammation may be helpful in distinguishing infectious from non-infectious complications in this specific group of patients [14].

## 2. Materials and Methods

Our observational study, which was not randomized, prospectively evaluated a cohort of 22 adult patients with lymphoproliferative neoplasms—mainly chronic lymphocytic leukemia (CLL) and a much smaller number with follicular lymphoma (FL)—with indications for therapy including obinotuzumab. The patients were treated at the Department of Hematology of the Military Medical Institute in Warsaw.

The study was approved by an ethics committee KB/1121/22, 12 December 2022. The purpose and procedures of the study were explained to the participants, and verbal and written informed consent were obtained. However, it should be emphasized that all patients qualified for the study had full indications for standard anti-CD20+ antibody treatment.

In particular, patients included in our observational study had indications for obinotuzomab therapy as a first-line therapy, according to the Polish national program for the treatment of chronic lymphocytic leukemia and follicular lymphomas.

General eligibility criteria: (1) age 18 or over; (2) performance status 0–2, according to the ECOG scale (Eastern Cooperative Oncology Group’s performance scale); (3) diagnosis of chronic lymphocytic leukemia; (4) presence of indications for treatment, according to the International Workshop on Chronic Lymphocytic Leukemia (the National Cancer Institute Working Group; IWCLL); (5) no contraindications to the drug, according to the current Summary of Product Characteristics; (6) absence of hypersensitivity to any of the drug or mouse proteins or any of the drug’s excipients; (7) exclusion of pregnancy and lactation; (8) patient consent to contraception in accordance with the relevant current Summary of Product Characteristics; (9) absence of active and serious infections; and (10) absence of significant comorbidities or clinical conditions contraindicating therapy.

Important exclusion criteria: (1) the occurrence of symptoms of hypersensitivity to any of the drugs used or to any of the drug excipients or to mouse proteins (Common Terminology Criteria for Adverse Events (CTCAE) grade 4 infusion-related reactions and recurrence of grade 2 infusion-related reactions), making it impossible to continue treatment; (2) period of pregnancy or lactation; and (3) the occurrence of diseases or conditions that, in the judgment of the treating physician, make it impossible to continue treatment.

All patients had their blood drawn for the first time to determine blood counts and CRP and procalcitonin values as inflammatory markers on day 0 before administration of the anti-CD20 antibody. In addition, all patients had their body temperature measured and blood cultures performed for bacterial infections. Subsequently, on days 1 to 7 after obinotuzumab administration, blood was drawn daily at a fixed time of 8:00 a.m. for blood counts and CRP and PCT values, and blood cultures were performed. In addition, on days 1 to 7, body temperature was measured at fixed times (i.e., 8:00 a.m. and 8:00 p.m.).

The ECOG scale (Eastern Cooperative Oncology Group’s performance scale), which allows for determination of the general condition and quality of life of a patient with cancer, was used to assess the clinical condition of patients. The Rai and Binet scales were used to assess the stage of chronic lymphocytic leukemia. To assess the stage of CLL, the Rai or Binet classifications were used, which distinguish the early (Rai 0; Binet A), intermediate (Rai I–II; Binet B), or advanced (Rai III–IV; Binet C) stages [15].

The procalcitonin test was performed using the RL-A2000 analyzer by Hangzhou Realy Tech Co., Ltd., Hangzhou, China for quantitative testing, where the laboratory standard range is 0.05–0.1 ng/mL. The CRP test was performed using a medical CRP analyzer PalmF by Hipro Shijazuang Hipro Biotechnology, Shijazuang, China, where the laboratory standard for CRP is 0.1–0.5 mg/dL. A SYSMEX XN-1500 analyzer from Sysmex Inc., Linconshire, IL, USA was used for blood counts, where the accepted laboratory standards are as follows: hemoglobin (Hb 12–16.5 g/dL), lymphocytes (0.8–3.5 G/L), neutrophils (1.8–6.5 G/L), platelets (130–350 G/L), and white blood cells (WBCs 4.0–10.0 G/L).

### Statistical Methodology

All statistical calculations were performed using the statistical package StatSoft. Inc. 2014. STATISTICA data analysis software system (version 12.0) by TIBCO Software Inc. Stanford Research Park, Palo Alto, CA, USA and an Excel spreadsheet. Quantitative variables were characterized according to the arithmetic mean, standard deviation median, minimum and maximum value range, and 95% confidence interval (CI). Qualitative variables were presented as counts and percentages. To test whether a quantitative variable came from a population with a normal distribution, the Shapiro–Wilk W test was used. The Levene–Brown–Forsythe test was used to test the hypothesis of equal variances. The significance of differences between two group uncorrelated variables model was tested using Student’s *t*-test or, if the variances were not homogeneous, the Welch test or the Mann–Whitney U-test if the conditions for the applicability of Student’s *t*-test were not met or for variables measured on an ordinal scale. The significance of differences between more than two groups was tested with the F-test ANOVA, or the Kruskal–Wallis test when the conditions of applicability of ANOVA were not met. When statistically significant differences between groups were obtained, post hoc tests were used for the F-test (Tukey test) or Kruskal–Wallis test (Dunn test). In the case of a model of two related variables, Student’s *t*-test or the Wilcoxon paired *t*-test was used when the conditions for applicability of Student’s *t*-test were not met or for variables measured on an ordinal scale. The significance of differences between more than two related variables in a model was tested via analysis of variance with repeated measures or the Friedman test when the conditions for applicability of analysis of variance with repeated measures were not met or for variables measured on an ordinal scale. Chi-square tests of independence were used for qualitative variables, using Yates correction for cell counts below 10, and checking Cochran’s conditions and Fisher’s exact test. In order to ascertain the association, strength, and direction between variables, correlation analysis was conducted by calculating Pearson and/or Spearman correlation coefficients. In all calculations, *p* = 0.05 was taken as the significance level.

## 3. Results

In the studied cohort, the percentages of women and men were 54.5% and 45.5%, respectively. The mean age of the patients was 70.5 (7.8) years old (range: 55–82 years old). The percentages of patients with CLL and FL diagnoses were 81.8% and 18.2%, respectively. According to the ECOG, the percentage of fully active patients (0) was 9.1%, those who were restricted in physically strenuous activity (1) accounted for 59.1%, and the percentage of those patients with ambulatory and self-care capabilities but who were unable to carry out any work activities (2) was 31.8%. The percentages of patients in the RAI classification stages (I–IV) were 11.1%, 44.4%, 27.8%, and 16.7%, respectively, while the percentages of patients in the BINET classification stages (A–C) were 5.5%, 55.6%, and 38.9%, respectively (see Table 1).

The mean WBC count in the investigated cohort was 99.8 (99.5) (range: 5.2–336.0), the mean neutrophil count was 5.1 (3.0) (range: 0.3–11.3), the mean lymphocyte count was 83.8 (89.6) (range: 1.5–320.4), the mean monocyte count was 9.7 (14.8) (range: 0.1–51.1), the mean platelet count was 84.0 (89.8) (range: 33.0–422.0), and the mean hemoglobin level was 11.2 (2.5) (range: 7.5–15.7); see Table 2.

The PCT and CRP values were measured on day 0 (before obinutuzumab administration) and on days 1, 2, 3, 4, 5, 6, and 7 after drug administration.

The initial PCT serum level 0 was 0.06 (0.05). The PCT serum level at measurement 1 was 35.67 (39.30), followed by measurement 2—25.48 (29.99); measurement 3—13.65 (13.71); measurement 4—6.97 (7.54); measurement 5—4.42 (3.81); and measurement 6—1.84 (1.91). At the final measurement, the value was 1.03 (1.07).

**Figure 1:**
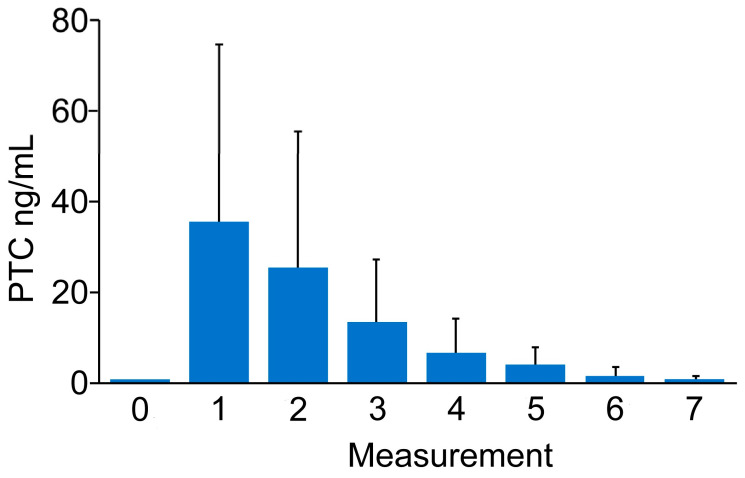
Course of PCT levels (ng/mL).

A statistically significant change in the PCT serum level was indicated with regard to the measurements (*p* < 0.0001). The PCT serum level was significantly increased in measurements taken on days 1 (*p* < 0.0001), 2 (*p* < 0.0001) 3 (*p* < 0.0001), and 4 (*p* = 0.0002), with regard to the initial measurement on day 0. Moreover, significant decreases in the PCT serum level on days 6 (*p* = 0.0032) and 7 (*p* < 0.0001) were observed in comparison with measurement 1. Similarly, significant decreases in the PCT serum level on days 6 (*p* = 0.0290) and 7 (*p* < 0.0006) were observed regarding measurement 2, while only a significant decrease in the PCT serum level on day 7 (*p* = 0.0365) was observed regarding measurement 3. No statistically significant changes were observed for the remaining comparisons; see Figure 1.

The initial CRP serum level on day 0 was 0.58 (0.85). The CRP serum level at measurement 1 was 6.45 (5.00), followed by measurement 2—1.82 (3.31); measurement 3—2.45 (5.94); measurement 4—3.93 (2.96); measurement 5—4.63 (3.00); and measurement 6—3.59 (1.91). In the final measurement, the value was 1.03 (1.07).

**Figure 2:**
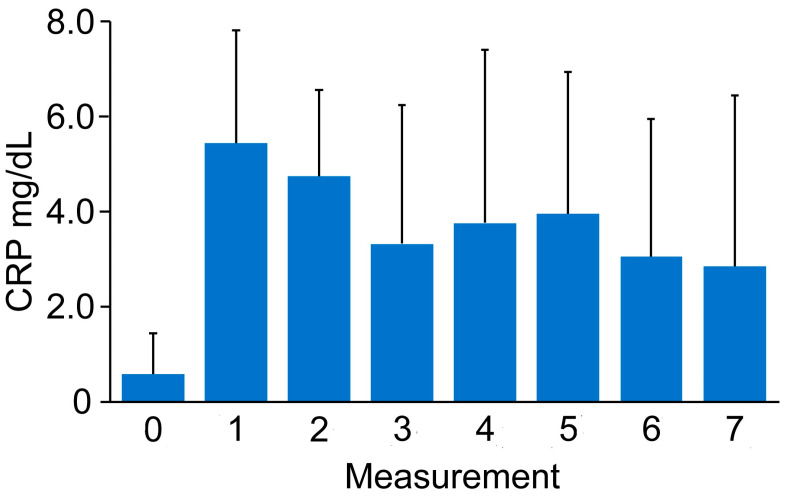
Course of CRP levels (mg/dL).

A statistically significant change in the CRP serum level was indicated regarding the obtained measurements (*p* < 0.0001). The CRP serum level was significantly increased in the measurements on days 1 (*p* < 0.0001), 2 (*p* < 0.0001), 3 (*p* < 0.0001), 4 (*p* = 0.0112), 5 (*p* = 0.0028), and 6 (*p* = 0.0266) regarding the initial measurement. Moreover, a significant increase in the CRP serum level on day 7 (*p* = 0.0088) was observed in comparison with measurement 1. No statistically significant changes were observed for the remaining comparisons; see Figure 2.

In the studied group, the percentages of normalization of the inflammatory markers on the seventh day and beyond the seventh day were 5.9% and 94.1%, respectively.

With an increase in the PCT serum level on day 0, a decrease in hemoglobin was observed (correlation coefficient R = −0.50; *p* = 0.0346). With an increase in the PCT serum level on day 1, increases in WBCs (correlation coefficient R = 0.50; *p* = 0.0280) and lymphocytes (correlation coefficient R = 0.50; *p* = 0.0306) were indicated.

This phenomenon may have been related to the fact that the higher the stage of the disease, the greater the likelihood of increased inflammatory parameters.

With an increase in the PCT serum level on day 0, increases in ECOG (correlation coefficient R = 0.53; *p* = 0.0243), RAI (correlation coefficient R = 0.56; *p* = 0.0234), and BINET (correlation coefficient R = 0.50; *p* = 0.0484) were noted. However, with an increase in the PCT serum level on day 5, a decrease in ECOG was observed (correlation coefficient R = 0.95; *p* = 0.0138).

With an increase in the CRP serum level on day 1, increases in the WBC count (correlation coefficient R = 0.44; *p* = 0.0407) and lymphocyte count (correlation coefficient R = 0.46; *p* = 0.0294) were noted, whereas the platelet count decreased (correlation coefficient R = −0.60; *p* = 0.0031). With an increase in the hemoglobin level, decreases in the CRP serum levels on days 4, 6, and 7 were observed (correlation coefficient R = −0.66, *p* = 0.0294; R = −0.66, *p* = 0.0070; R = −0.67, *p* = 0.024, respectively). With an increase in the CRP serum level on day 7, decreases in the neutrophil count (correlation coefficient R = −0.53; *p* = 0.0251) and platelet count (correlation coefficient R = −0.49; *p* = 0.0383) were observed.

The observed situation was related to the patients’ general condition; that is, the worse the general condition, the greater the likelihood of a significant increase in inflammatory indices. In the patients, we did not observe an increase in body temperature in any of the patients, nor did bacteriological studies of blood samples reveal an increase in bacterial cultures.

## 4. Discussion

In various patient populations, PCT and CRP have both been demonstrated to be effective in identifying systemic inflammation. It has been proposed that PCT is more significant than other markers of infection for patients with neutropenic fever. A distinct group of patients with a markedly changed immune response are those receiving conditioning therapy before receiving a hematopoietic stem cell transplant. This makes it harder to clinically diagnose early infections, which can have disastrous consequences if not caught in time. ATG is a complicated protein, and its use may result in negative side effects—primarily respiratory failure and/or circulatory instability. According to our studies, the intensity of these reactions can vary greatly from person to person, occasionally mimicking sepsis. In this regard, it would be very beneficial to have more biochemical tests that might quickly identify infections. We observed a typical early increase in PCT and CRP levels in our group of patients treated with obinutuzumab, followed by a slow decrease to values that were nearly normal. Microbiological methods were used to track this event; however, it was not linked to any clinical infections.

Therefore, after using an immunosuppressive monoclonal antibody, neither PCT nor CRP were observed to be effective as trustworthy diagnostic techniques in the context of possible infection. It is important to remember that immunosuppressive medications may make serious infections more likely. The literature provides some support for our observations: prior research has indicated that PCT and CRP have little diagnostic use when T-cell antibodies are present. Sabat et al. found that pan-T-cell antibodies increased PCT levels in renal transplant recipients in a manner similar to that of sepsis [16]. An early increase was observed 24 h following the initiation of therapy, which is consistent with our findings. Within minutes of administering ATG, an increase in tumor necrosis factor alpha (TNF-α) levels was observed, preceding the increase in the PCT level. However, these patients showed no symptoms of infection. Similarly, Zazula et al. found that patients receiving orthotopic liver transplantation had higher PCT levels the day following surgery, with the increase being more noticeable in those who received ATG.

In both patient groups, PCT levels dropped without further ATG dosing, and neither group showed any symptoms of infection [17].

In a short retrospective investigation, Dornbusch et al. assessed the diagnostic utility of PCT and CRP in distinguishing between febrile responses and sepsis following the administration of anti-T-cell antibodies in pediatric patients. Patients with sepsis and those receiving T-cell antibodies did not differ in their highest PCT levels or concentrations three days after the onset of febrile responses [18]. Following stem cell transplantation, 350 patients were prospectively followed-up with by Pihusch et al. for PCT, CRP, and interleukin-6 (IL-6). They provided evidence that ATG is not the only factor contributing to elevated levels of PCT and CRP [19]. Following therapy with different T-cell antibodies, such as OKT-3, comparable results were observed. Higher levels of PCT and CRP were produced after treatment with the CD52 monoclonal antibody alemtuzumab, similar to the sepsis brought on by Gram-negative bacteria. We still do not fully understand the precise role, method, and manufacturing location of PCT, and human leukocytes have been shown to exhibit PCT-related activities. Other researchers have proposed that its manufacturing may occur in the liver, lungs, neuroendocrine cells, or other tissues. It has also been noted that TNF-α significantly stimulates PCT mRNA levels or PCT itself [20,21,22].

Emlanasser et al. reported a case of serum sickness (SS) reaction after obinotuzumab administration. Their patient presented the full symptoms of this reaction, in the form of a maculopapular rash, fever, and polyarthritis, as well as elevated inflammatory markers [23,24]. Among all the patients we observed, there was not a single suspected case of serum sickness reaction, as all of our patients had an immediate reaction rather than the symptoms that usually appear 1–2 weeks after exposure to the antibody [24]. In our observational study, the lack of positive blood cultures and the absence of an increase in body temperature in the observed patients support the fact that the increase in inflammatory exponents must be directly related to the administered drug.

A limitation of our study is that it only included a small group of study subjects, as we only observed patients treated at our center. It would have been useful to have gathered a larger group of subjects and considered more inflammatory cytokines. In the future, we will certainly continue our observations and expand their scope to include the behaviors of pro- and anti-inflammatory interleukins. Nevertheless, even with this small group of patients, significant increases in the values of inflammatory exponents were clearly observed, even on the very next day after the administration of the anti-CD20 antibody, which can often—in the absence of this knowledge—lead to the unnecessary implementation of antibiotic therapy. Our study proves that the increases in inflammatory proteins are not related to infections.

## 5. Conclusions

In conclusion, while an ideal biomarker does not exist, procalcitonin is undoubtedly one of the best markers currently available for the early detection of infection risk. While PCT has high sensitivity and specificity in infections, and rapidly increases at the onset of infection, it also has certain limitations; for example, it also increases in value under conditions unrelated to infections. In our study, we showed that increases in procalcitonin and CRP values occur very quickly, in virtually every case, following the administration of obinutuzumab. Based on our observations, it seems that even very large increases in these levels do not indicate that treatment is required, and the situation generally resolves spontaneously. Furthermore, it does not lead to any adverse sequelae. However, an increase in procalcitonin must always be taken into account as an indicator of a possible severe infection; as such, we must also pay attention to other signs of infection in addition to an increase in procalcitonin or CRP.

## Figures and Tables

**Table 1:** Fundamental features of the investigated cohort regarding gender, age, diagnosis, ECOG performance status scale, and clinical staging of the disease according to RAI and BINET classifications.

	Cohort (*n* = 22)
**Sex**	
female	12 (54.5%)
male	10 (45.5%)
**Age**	
mean	70.5 (7.8)
range	55.0–82.0
median	72.0 (10.0)
95%CI	[67.0; 74.0]
**Diagnosis**	
CLL	18 (81.8%)
FL	4 (18.2%)
**ECOG**	
0	2 (9.1%)
1	13 (59.1%)
2	7 (31.8%)
**RAI**	
I	2 (11.1%)
II	8 (44.4%)
III	5 (27.8%)
IV	3 (16.7%)
**BINET**	
A	1 (5.5%)
B	10 (55.6%)
C	7 (38.9%)

**Table 2:** Characteristics of the investigated group regarding WBCs, neutrophils, lymphocytes, monocytes, platelets, and hemoglobin.

	Cohort (*n* = 22)
**White blood cells (WBCs) (G/L)**	
mean (SD)	99.8 (99.5)
range	5.2–336.0
median (IRQ)	73.7 (138.7)
95%CI	[55.7; 143.9]
**Neutrophils (G/L)**	
mean (SD)	5.1 (3.0)
range	0.3–11.3
median (IRQ)	4.1 (4.4)
95%CI	[3.7; 6.4]
**Lymphocytes (G/L)**	
mean (SD)	83.8 (89.6)
range	1.5–320.4
median (IRQ)	58.2 (121.9)
95%CI	[44.1; 123.6]
**Platelets (G/L)**	
mean (SD)	184.0 (89.8)
range	33.0–422.0
median (IRQ)	161.0 (120.0)
95%CI	[144.2; 223.8]
**Hemoglobin (Hb) (g/dL)**	
mean (SD)	11.2 (2.5)
range	7.5–15.7
median (IRQ)	11.9 (4.9)
95%CI	[10.1; 12.3]

## Data Availability

The data presented in this study are available in this article, and further inquiries can be directed to the corresponding author.
